# Isobutyric Acid Promotes Immune Evasion in Colorectal Cancer via Increased PD‐L1 Expression

**DOI:** 10.1002/cam4.70397

**Published:** 2024-11-06

**Authors:** Qiuhua Lin, Han Wang, Wenbo Chen, Xinjie Wei, Jinglian Chen, Ying Deng, Chunyin Wei, Hao Lai, Xianwei Mo, Weizhong Tang, Tao Luo

**Affiliations:** ^1^ Department of Gastrointestinal Surgery Guangxi Medical University Cancer Hospital, Guangxi Medical University Nanning Guangxi P.R. China; ^2^ Guangxi Key Laboratory of Basic and Translational Research of Colorectal Cancer Nanning Guangxi P.R. China; ^3^ Department of Ultrasound Guangxi Medical University Cancer Hospital, Guangxi Medical University Nanning Guangxi P.R. China

**Keywords:** colorectal cancer, immune escape, isobutyric acid, PD‐L1, ROCK1

## Abstract

**Introduction:**

Isobutyric acid (IBA), a short‐chain fatty acid, has been unequivocally demonstrated to exert significant influence on the progression of colorectal cancer (CRC). Nevertheless, a comprehensive understanding of its intricate regulatory mechanisms remains elusive.

**Methods:**

Employing advanced techniques such as western blot, RT‐qPCR, and flow cytometry, we systematically investigated the impact of IBA on the expression of PD‐L1 in CRC cells. Concurrently, employing RNA silencing technology and small‐molecule inhibitors, we delved into the molecular intricacies underlying the regulatory axis of IBA involving ROCK1/c‐Myc/PD‐L1. Furthermore, through flow cytometry analysis, we examined the alterations in the tumor immune microenvironment following anti‐PD‐L1 antibody therapy in a murine tumor model treated with IBA.

**Results:**

Elevated levels of IBA were found to robustly activate PD‐L1 expression in CRC cells both in vitro and in vivo, concomitantly reshaping the tumor immune microenvironment. Subsequent mechanistic investigations unveiled that IBA, through its interaction and activation of ROCK1, promotes the activation of c‐Myc, thereby enhancing the transcription of PD‐L1. Silencing of ROCK1 and application of ROCK1 inhibitors effectively reversed the regulatory effects of IBA on PD‐L1. Additionally, IBA inhibited the activity of infiltrating CD8^+^ T cells, resulting in diminished antitumor immunity and attenuating the sensitivity to anti‐PD‐L1 therapy.

**Conclusion:**

Our study elucidates a novel mechanism by which IBA inhibits the sensitivity of CRC to anti‐PD‐L1 antibody therapy. Emphasizing IBA and its downstream pathways as potential therapeutic targets for immune therapy resistance mechanisms, our findings provide a novel theoretical foundation for overcoming immune therapy resistance.

## Graphical abstract

Isobutyric acid (IBA) significantly influences colorectal cancer (CRC) progression by activating PD‐L1 expression through the ROCK1/c‐Myc axis. Additionally, IBA reshapes the tumor immune microenvironment, inhibiting CD8^+^ T cells and thereby reducing sensitivity to anti‐PD‐L1 therapy. Targeting the IBA/ROCK1/c‐Myc/PD‐L1 pathway may offer a novel approach to overcoming immune therapy resistance in CRC.

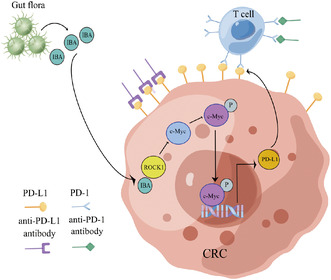

**Abbreviations**
CRC: colorectal cancer
CTL: cytotoxic T lymphocyte
IBA: isobutyric acid
ICI: immune checkpoint inhibitor
MSI: microsatellite instability
MSS: microsatellite stability
TIL: tumor‐infiltrating lymphocytes
TMB: tumor mutation burden.

## 1 Introduction

Colorectal cancer (CRC) constitutes one of the malignant neoplasms that gravely jeopardize human health on a global scale [1]. In recent years, immunotherapy has emerged as a critical modality for treating various tumors, particularly through the activation of immune cells by immune checkpoint inhibitors (ICIs), significantly enhancing therapeutic efficacy in oncology [2]. The application of inhibitors targeting the programmed cell death protein 1 (PD‐1)/programmed death‐ligand 1 (PD‐L1) pathway is notably widespread. PD‐L1 is extensively expressed across a variety of cells, predominantly encompassing tumor cells and those within the tumor microenvironment [3]. Inhibitors of the immune checkpoint PD‐1/PD‐L1 axis disrupt the interaction between PD‐1 on T cells and PD‐L1 on tumor or stromal cells, augmenting the antitumor activity of CD8^+^ cytotoxic T lymphocytes (CTLs) [4]. This offers a beacon of hope for patients with refractory malignancies.

Based on DNA mismatch repair gene mutations, CRC can be categorized into microsatellite instability (MSI) and microsatellite stability (MSS) subtypes [5]. MSI CRCs, accounting for only 12%–15% of all cases, are characterized by a higher tumor mutation burden (TMB) and the presence of tumor‐infiltrating lymphocytes (TILs), enabling a subset of patients to benefit from ICIs therapy [6]. In contrast, the predominant MSS CRC exhibit minimal or virtually no response to monotherapy with PD‐1/PD‐L1 ICIs [7], significantly constraining the clinical application of immune checkpoint blockade therapy in CRC. Consequently, delving into the mechanisms of resistance to immunotherapy is essential.

It is widely recognized that metabolites within the body can modulate the tumor microenvironment, with an increasing number of findings revealing the impact of gut microbiota metabolites on cancer, including their effects on intestinal and immune system functions [8, 9, 10]. However, the mechanisms underlying these effects remain to be elucidated. Isobutyric acid (IBA) is produced by anaerobic bacteria in the gut microbiota through the fermentation of valine, representing a final product of bacterial amino acid metabolism [11, 12]. Despite numerous studies indicating that the concentration of IBA in CRC patients is significantly elevated compared to healthy individuals [13], the potential role of IBA in CRC and its impact on sensitivity to immunotherapy remain unknown.

This study is dedicated to investigating the impact of IBA on the immune therapeutic response in CRC. Our findings reveal that IBA enhances the expression of PD‐L1 in CRC, manifested at both the protein and mRNA levels, and both at the cellular and animal levels. This is reflected in the promotion of tumor growth and the reshaping of the tumor immune microenvironment. Furthermore, we discovered that IBA, through its binding to ROCK1 and subsequent activation, facilitates the activation of the downstream transcription factor c‐Myc, thereby augmenting the transcription of PD‐L1. In summary, our research has for the first time deciphered the subtle molecular characteristics behind IBA's role in resistance to immunotherapy in CRC. This not only broadens our comprehension of CRC but also aids in the formulation of targeted therapeutic strategies.

## 2 Materials and Methods

### 2.1 Patients and Specimens

Tumor tissue samples were obtained from 87 patients with CRC who underwent surgery at the Guangxi Medical University Cancer Hospital from January 2019 to December 2021. All patients included in this study underwent curative CRC resection, with a pathological diagnosis of adenocarcinoma. None of them had received preoperative radiotherapy or chemotherapy. Ethical approval for this study was granted by the Ethics Committee of the Guangxi Medical University Cancer Hospital. Written informed consent for the use of their specimens and information was obtained from all patients.

### 2.2 Cell Culture

Human CRC cell lines SW480, HT29, and SW620, human embryonic kidney cells HEK293T, and mouse CRC cell lines CT26 and MC38 were obtained from the American Type Culture Collection (Manassas, Virginia, USA) and the National Infrastructure of Cell Line Resource (Beijing, China), respectively. Cell lines were cultured in their respective media supplemented with 10% fetal bovine serum (FBS, Gibco, CA, USA) and a mixture of 100 U/mL penicillin and 100 μg/mL streptomycin, and incubated at 37°C in a humidified incubator with 5% CO_2_.

### 2.3 Plasmid Construction

Plasmid construction was carried out using standard molecular cloning techniques. The c‐Myc gene was cloned into the pcDNA3.1 vector by PCR amplification of the full‐length c‐Myc cDNA and its subsequent insertion into the pcDNA3.1 vector.

### 2.4 Transfections and Viral Infections

Transfection of 80% confluent cells with Lipofectamine 2000 Reagent (Invitrogen, USA) was performed in Opti‐MEM medium (Invitrogen, USA). HEK293T cells were employed for packaging the silenced lentivirus, which was subsequently used to infect CRC cells. After 48 h of infection, cells were subjected to a 3‐day selection process with puromycin (2 μg/mL). The effectiveness of virus infection was assessed through western blot analysis.

### 2.5 Quantitative Polymerase Chain Reaction (qPCR)

According to the manufacturer's protocol, total RNA was isolated from cell lines using TRIzol reagent (Invitrogen, CA, USA). The purity and integrity of RNA were assessed by measuring the absorbance ratio at 260 nm/230 nm using a microvolume spectrophotometer and by detecting the intensity of the 28S and 18S rRNA bands through agarose gel electrophoresis. RNA meeting the specified requirements was then reverse transcribed into cDNA using the PrimeScript RT reagent kit with gDNA eraser (Takara, Dalian, China). Real‐time qPCR (RT‐qPCR) was performed using the FastStart Universal SYBR Green Master (ROX) reagent kit (Roche, Germany) to assess the expression of RNA. The relative expression levels were normalized to the endogenous control (GAPDH) using the relative quantification method (2^−ΔΔCt^). The primer sequences are delineated in Table S1.

### 2.6 Western Blot

In accordance with previous studies [14], cell lysis was achieved by supplementing NP‐40 buffer (Beyotime, China) with protease inhibitors (MedChemExpress, USA) and phosphatase inhibitors (MedChemExpress, USA) on ice, ensuring a comprehensive disruption of cellular components. The concentration of the protein extracts was determined following thorough lysis on ice. Subsequently, 20 μg of protein was subjected to 10%–12% SDS‐PAGE electrophoresis and transferred onto nitrocellulose membranes. Following blocking with 5% skimmed milk in Tris‐buffered saline, the membranes were washed and incubated with specific antibodies. The primary antibodies were diluted in 5% BSA in TBST, and the secondary antibodies were diluted in 5% skimmed milk.

### 2.7 In Vitro Pull‐Down Assay

For the pull‐down assay, a methodology consistent with previous investigations was employed [13]. In essence, 0.5–1 mg of protein lysate was incubated with appropriate magnetic beads at 4°C for 4 h. Following this, the immunocomplexes were washed five times with NP‐40 buffer before resolving the immune complexes through SDS‐PAGE and subsequently subjecting the indicated proteins to western blot analysis.

### 2.8 Flow Cytometry

Following digestion and mincing of tumor tissues with collagenase, red blood cells within were lysed, then cultured at 37°C for 8 h in medium containing Cell Stimulation Cocktail (1:500, eBiosciences). For cell surface staining, cells were stained with Zombie, anti‐CD45, and anti‐CD8 antibodies (1:100, Biolegend). For intracellular staining, cells were treated with Fixation/Permeabilization Solution Kit (Invitrogen), and stained with anti‐IFN‐γ and anti‐Granzyme B antibody (1:100 dilution, Biolegend). Additionally, at the end of cellular experiments, cells were digested with trypsin without EDTA and stained with anti‐PD‐L1 antibody (1:100 dilution, Biolegend). Data were acquired on a CytoFLEX (Beckman Coulter, USA) and analyzed using FlowJo software (TreeStar Inc., USA).

### 2.9 In Vivo Experiment in Mice Tumor Models

Animal studies were approved by the Animal Ethics Committee of Guangxi Medical University, and female BALB/c, C57BL/6J, and BALB/c nude mice aged 4–6 weeks were procured from the Experimental Animal Center of Guangxi Medical University. Consistent with previous research [13], IBA (200 mM, Sigma, USA) was added to the drinking water of mice 4 weeks prior to treatment. To establish tumor models, we subcutaneously injected 1 × 10^5^ CT26 or MC38 cells in 100 μL of PBS into the abdominal skin of BALB/c or C57BL/6J mice, respectively. Likewise, LoVo cells were injected into BALB/c nude mice using a similar procedure. For systemic treatment with Y27632 (10 mg/kg, MCE, USA) or anti‐PD‐L1 mAb (200 μg/mouse, Bio X Cell, USA), intraperitoneal injections commenced 1 week postsubcutaneous tumor inoculation. Tumor volumes were calculated using the formula length × width [2] × 0.5.

### 2.10 Immunohistochemistry Analysis

Subcutaneous CRC tumor tissues were fixed in formalin, dehydrated, embedded in paraffin, and sectioned for conventional immunohistochemical staining. The primary antibodies used included PD‐L1 (IHC, 1:100, ABclonal, CN), ROCK1 (IHC, 1:100, ABclonal, CN), c‐Myc (IHC, 1:100, Maxim, CN), and CD8 (IHC, 1:100, Abcam, CN).

### 2.11 Cellular Thermal Shift Assay

IBA or DMSO was incubated with HEK293T cell lysate supernatants at room temperature for 1 h. Following incubation, the lysates were aliquoted into several tubes (50 μL per tube) and then subjected to heating at various temperatures for 5 min. After cooling at room temperature for 3 min, the samples were centrifuged at 4°C and 13000*g* for 20 min. The supernatants were collected and subsequently heated in a metal bath at 100°C for 5 min. Finally, protein stability was assessed through SDS‐PAGE electrophoresis and western blot analysis.

### 2.12 Cell Proliferation Assay

CRC cell growth was assessed through EdU and CCK‐8 assays. In brief, CRC cells in logarithmic growth phase were digested with trypsin, and 10^4^ cells/well were seeded in a 96‐well plate. Various concentrations of IBA were added to the culture medium containing 10% FBS for continued cultivation. Subsequently, cell growth was evaluated according to the manufacturer's instructions through EdU and CCK‐8 assays.

### 2.13 ROCK Kinase Activity Assay

Following treatment of CRC cells in culture with either IBA (5 mM) or Y27632 (20 μM) for 48 h, the cells were lysed using NP‐40 buffer, and the supernatant containing proteins was obtained through centrifugation. The kinase activity of ROCK1 was determined in accordance with the protocol of the Cellular ROCK1 Kinase Activity Assay Kit (GENMED Scientific INC., USA).

### 2.14 Statistical Analysis

Figures and statistical analyses were conducted utilizing GraphPad Prism 7 (La Jolla, CA, USA) and SPSS 22.0 (Chicago, IL, USA). The presented data are expressed as the mean ± standard error of the mean (SEM). For comparisons of quantitative and categorical data, the two‐tailed Student's *t*‐test and chi‐square test were employed, respectively. Pearson correlation coefficient was employed for correlation analyses. Specific statistical tests were applied and corresponding *p* values are delineated in each figure legend. A significance threshold of *p* < 0.05 was considered indicative of statistical significance.

## 3 Results

### 3.1 IBA Elicits PD‐L1 Expression in CRC Cells

**FIGURE 1:**
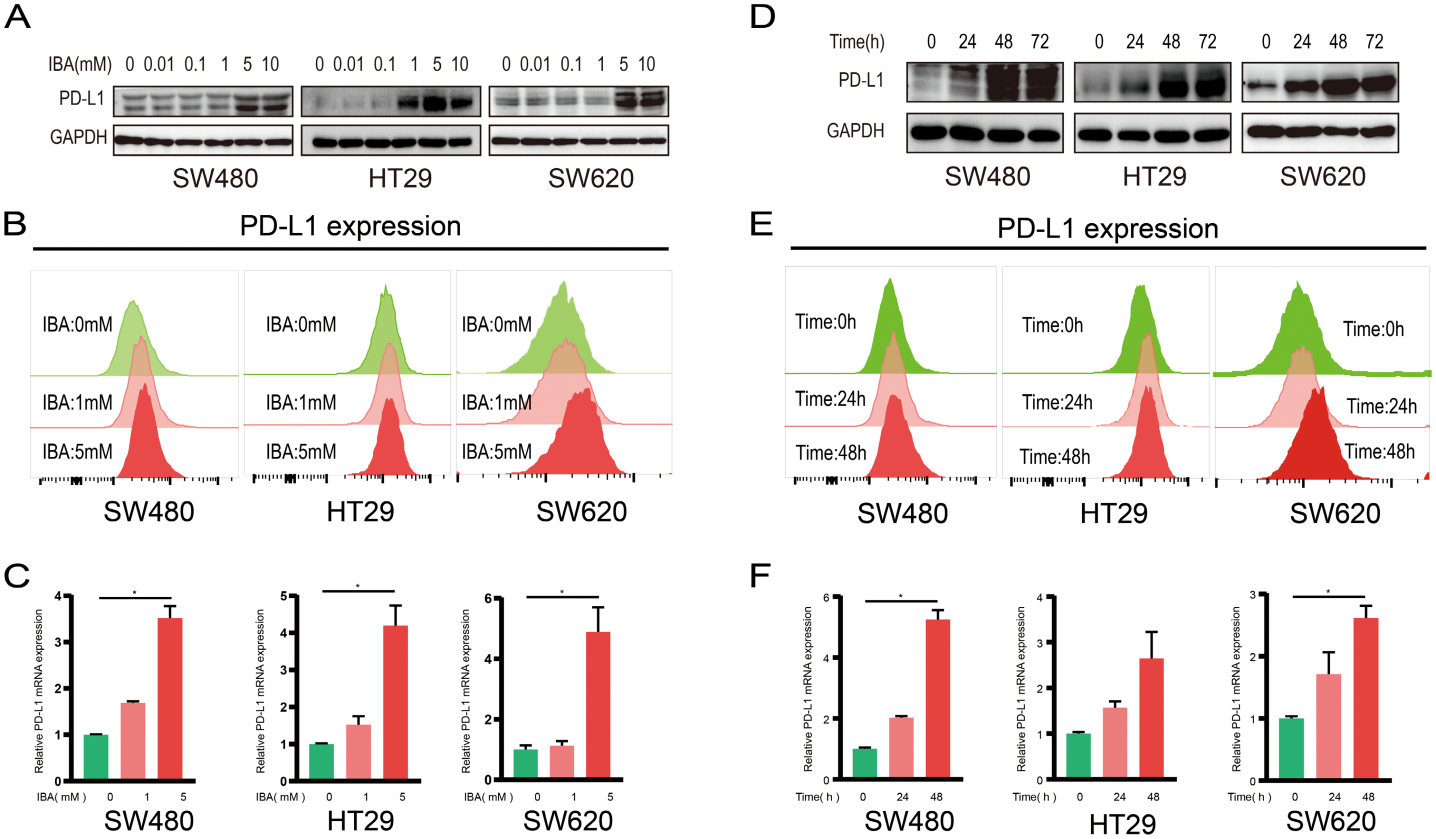
IBA Elicits PD‐L1 expression in CRC cells. (A–C) CRC cells were stimulated with specified concentrations of IBA for 48 h, and the expression of PD‐L1 was assessed through western blot (A), flow cytometry (B), and RT‐qPCR (C). (D–F) CRC cells were cultured at a concentration of 5 mM IBA for various durations, and the expression of PD‐L1 was evaluated through western blot (D), flow cytometry (E), and RT‐qPCR (F). NS, *p* ≥ 0.05. *, *p* < 0.05.

Aberrant expression of PD‐L1 constitutes one of the principal reasons for resistance to PD‐1/PD‐L1 inhibitor therapy [15]. To confirm whether IBA regulates PD‐L1 in CRC cells, CRC cells were treated with varying concentrations of IBA and the expression of PD‐L1 was assessed through western blot analysis. CRC cells treated with IBA exhibited elevated levels of PD‐L1 (Figure 1A). This phenomenon was similarly evident in flow cytometry and RT‐qPCR analyses (Figure 1B,C). Subsequently, we further subjected CRC cell lines to treatment with the same concentration of IBA, harvesting them at various time points to assess PD‐L1 levels. The results suggest a positive correlation between the duration of IBA exposure and the impact on PD‐L1 expression (Figure 1D–F). In conclusion, IBA upregulates PD‐L1 expression in CRC cells in a dose‐dependent and time‐dependent manner.

### 3.2 IBA Upregulates PD‐L1 Expression and Stimulates CRC Growth In Vivo

**FIGURE 2:**
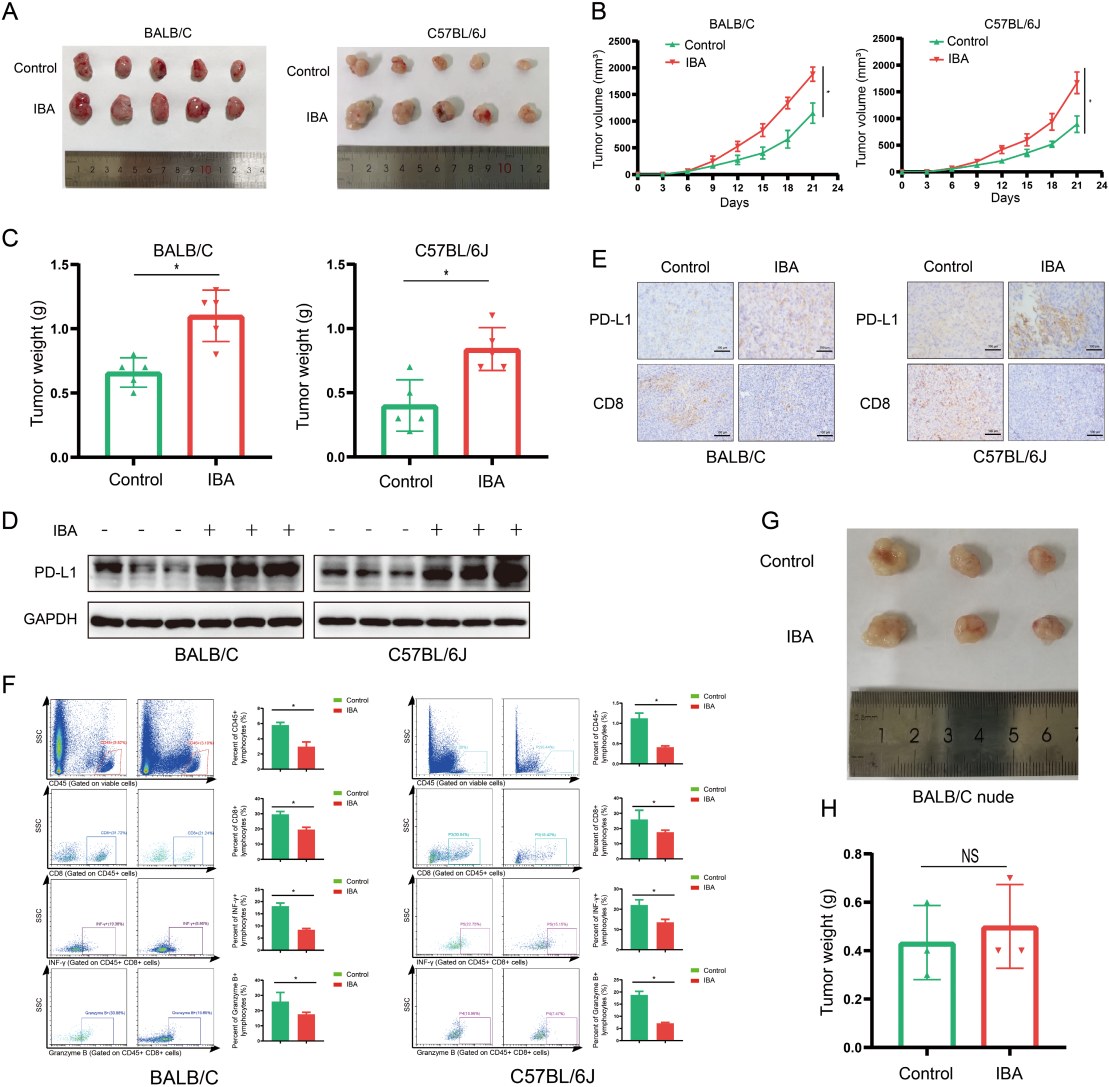
IBA upregulates PD‐L1 expression and stimulates CRC growth in vivo. (A–F) Subcutaneous syngeneic mouse model of CRC, where CT26 or MC38 cells were injected into BALB/c or C57BL/6J mice, respectively, including images of tumor tissue (A), tumor growth curve (B), and tumor mass (C) (*n* = 5). (D) Western blot assessing the expression of PD‐L1 in mouse tumor tissues. (E) IHC analysis of PD‐L1 and CD8 expression levels in mouse CRC tumors. Scale bar = 100 μm. (F) Flow cytometry analysis of CD45, CD8, IFNγ, and granzyme B expression in mouse CRC tissues. (G, H) Subcutaneous xenograft mouse model of CRC, where LoVo cells were injected into BALB/c nude mice, including images of tumor tissue (G) and tumor mass (H) (*n* = 3). NS, *p* ≥ 0.05. *, *p* < 0.05.

Given that IBA enhances the expression of PD‐L1 in CRC cells, it is imperative to elucidate the impact of IBA on PD‐L1 expression in CRC in vivo. Referring to previous studies, we successfully established a high IBA mouse model (Figure S1A,B) [13]. Subsequently, mouse CRC cells were subcutaneously injected into the high IBA mouse model, with a control group established. Strikingly, the visual results demonstrated significantly faster subcutaneous tumor growth in the high IBA group mice (Figure 2A–C). Western blot and IHC analyses confirmed that IBA upregulates PD‐L1 levels in CRC in vivo (Figure 2D,E). More importantly, IBA demonstrated an inhibitory effect on TILs within the tumor tissue (Figure 2E,F). Further validation revealed that IBA did not significantly affect the growth of subcutaneous tumors in BALB/c nude mice, which lack functional thymuses and T lymphocytes (Figure 2G,H). Similarly, the proliferation of human and mice CRC cells in vitro in the absence of T cells was not markedly influenced by IBA (Figure S2A–C). In summary, we have demonstrated within a CRC mouse model that IBA activates PD‐L1 expression in CRC, inhibits T lymphocyte activation, and facilitates tumor immune escape.

### 3.3 IBA Modulation of PD‐L1 is Closely Tied to c‐Myc Signaling

**FIGURE 3:**
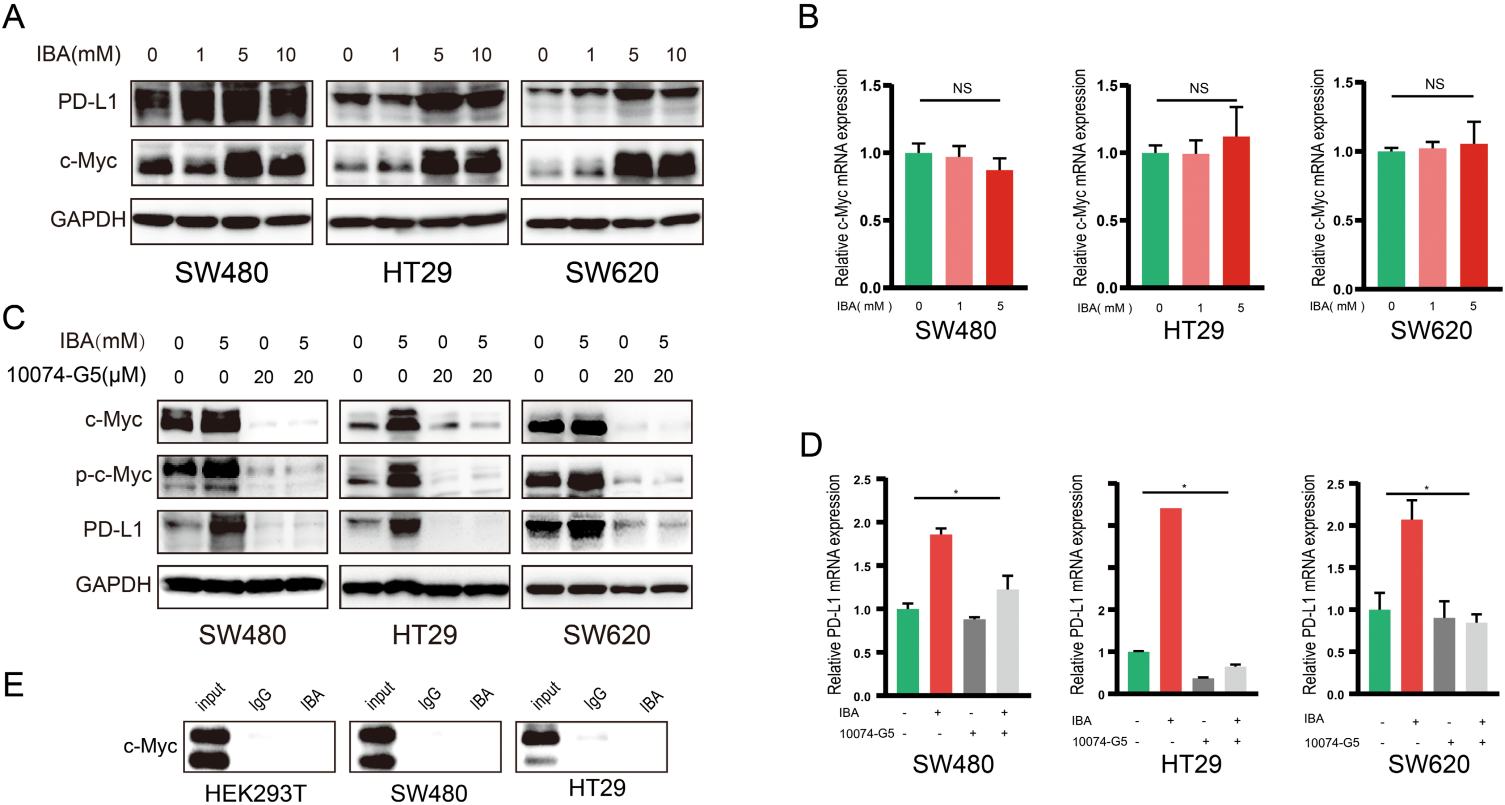
IBA modulation of PD‐L1 is closely tied to c‐Myc signaling. (A, B) CRC cells were treated with a specified concentration of IBA for 48 h, and alterations in c‐Myc protein (A) and mRNA (B) were assessed using western blot and RT‐qPCR, respectively. (C, D) CRC cells were co‐incubated with IBA and 10,074‐G5 for 48 h, and changes in c‐Myc protein (C) and mRNA (D) were evaluated through western blot and RT‐qPCR, respectively. (E) In vitro pull‐down assay was employed to scrutinize the interaction between IBA and c‐Myc. Data in (B) and (D) represent the mean ± SD of three independent experiments. NS, *p* ≥ 0.05. *, *p* < 0.05.

c‐Myc ranks among the pivotal transcription factors that directly regulate the expression of PD‐L1, possessing the capacity to bind to the promoter of *CD274*, which is the gene encoding PD‐L1, thereby directly activating its transcription [16, 17]. We hypothesized whether IBA regulates PD‐L1 expression through c‐Myc. Through western blot analysis, we discovered that IBA not only upregulates PD‐L1 protein expression in CRC cells but also enhances the expression of c‐Myc protein (Figure 3A). However, RT‐qPCR showed that IBA did not affect the level of c‐Myc mRNA (Figure 3B), suggesting that the impact of IBA on c‐Myc is primarily at the protein level rather than the transcriptional level. Subsequently, we treated CRC cells with both IBA and a c‐Myc inhibitor (10074‐G5) simultaneously and found that 10074‐G5 reversed the upregulation effect of IBA on PD‐L1, both at the protein level (Figure 3C) and mRNA level (Figure 3D). The cotreatment of CRC cells with IBA and the c‐Myc overexpression plasmid resulted in a markedly pronounced upregulation of PD‐L1 (Figure S3). Indeed, we discovered that IBA did not directly bind to endogenous c‐Myc protein (Figure 3E). Therefore, we propose that IBA regulates PD‐L1 through c‐Myc, possibly through an indirect mechanism.

### 3.4 IBA Emerges as the Physiological Activator for ROCK1

**FIGURE 4:**
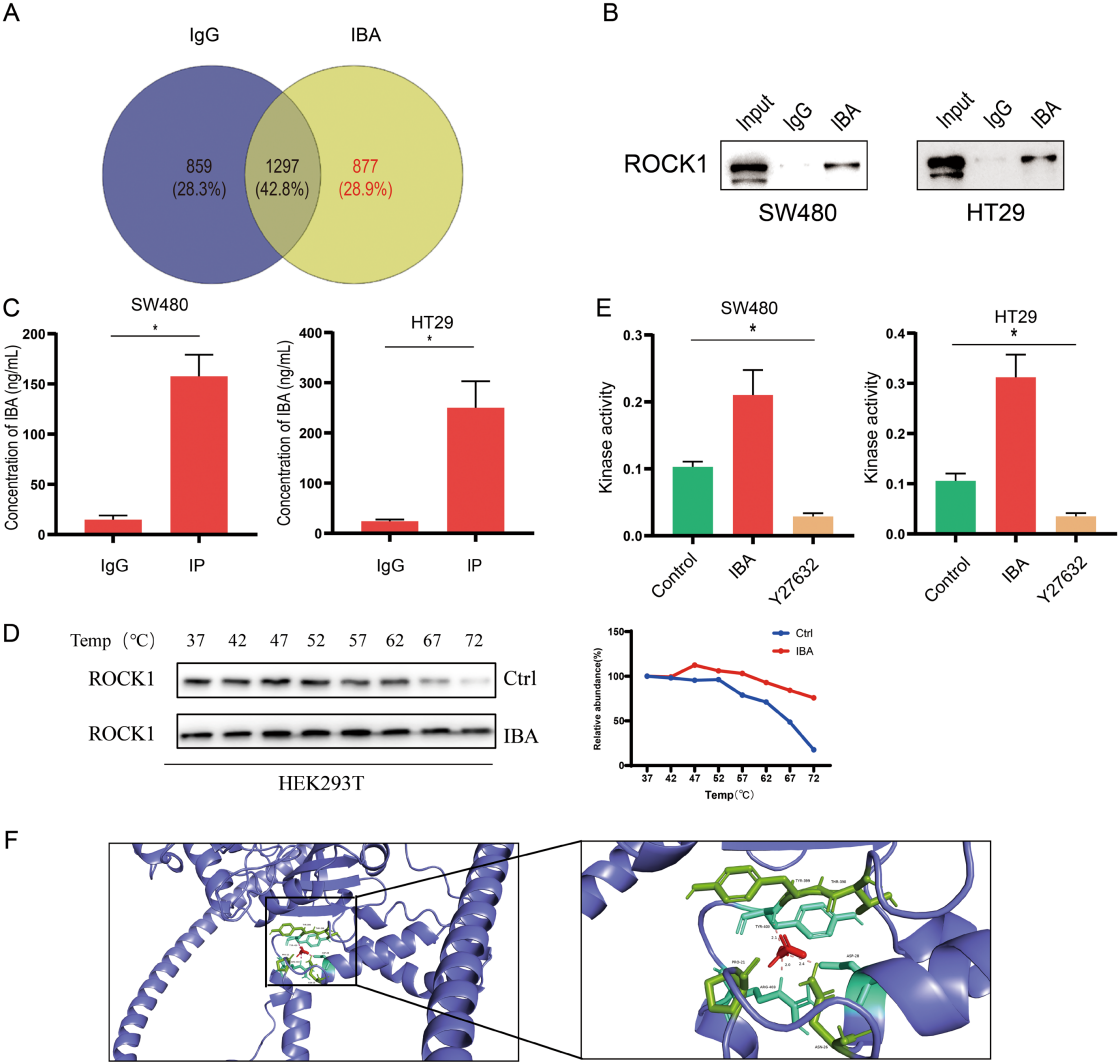
IBA emerges as the physiological activator for ROCK1. (A) MS analysis illustrating the specific binding proteins of IBA in a Venn diagram. (B) Validation of the specific binding of IBA to ROCK1 using an in vitro pull‐down assay and western blot analysis. (C) Detection of IBA concentration using LC–MS (*n* = 3); (D) Cellular thermal shift assay to assess the impact of IBA on the stability of ROCK1. (E) Stimulation of CRC cells for 48 h with IBA and Y27632 at concentrations of 5 mM and 20 μM, respectively, followed by the measurement of ROCK kinase activity (*n* = 3). (F) Schematic representation predicting the structural domains of ROCK1 binding to IBA. Data in (C and E) represent the mean ± SD of three independent experiments. *, *p* < 0.05.

To elucidate the potential binding partners of IBA, we initially executed an in vitro pull‐down assay to segregate IBA‐associated proteins from HEK293T cells, subsequently subjecting them to mass spectrometry analysis, in alignment with preceding research endeavors [13]. Our findings indicated that ROCK1 was among the potential binding proteins for IBA (Figure 4A). Existing research indicates that ROCK1 plays a pivotal role in facilitating tumor progression [18]. We hypothesized whether ROCK1 could mediate IBA's regulatory effects on PD‐L1. We discovered that IBA can bind to endogenous ROCK1 in CRC cells (Figure 4B), with ROCK1 proteins captured based on ROCK1 antibodies also exhibiting IBA binding (Figure 4C). The cellular thermal shift assay suggested that ROCK1 protein incubated with IBA exhibited enhanced stability (Figure 4D). In the ROCK1 kinase activity assay, the ROCK1 inhibitor Y27632 served as a positive control by inhibiting ROCK1 activity, whereas IBA was found to activate ROCK1 activity (Figure 4E). Additionally, the crystal structure of the ROCK1 kinase domain that potentially interacts with IBA was presented through macromolecular docking (Figure 4F). In conclusion, our findings demonstrate that IBA has the ability to bind to and activate ROCK1.

### 3.5 IBA‐Induced PD‐L1 Expression Hinges on the ROCK1/c‐Myc Signaling Axis in CRC

**FIGURE 5:**
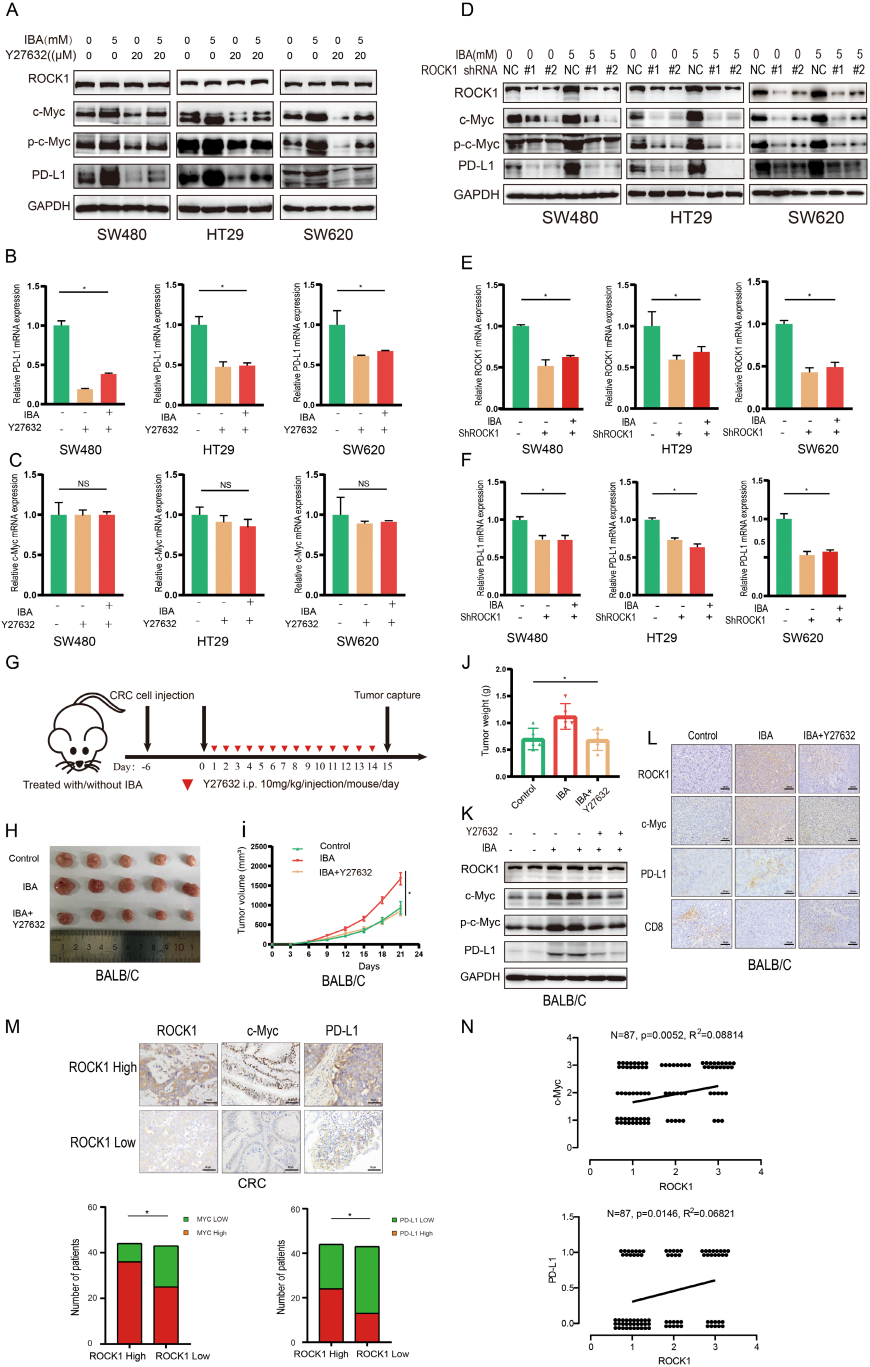
IBA‐induced PD‐L1 expression hinges on the ROCK1/c‐Myc signaling axis in CRC. (A‐C) CRC cells were incubated with IBA and Y27632 for 48 h, and the expression levels of PD‐L1 and c‐Myc were assessed through western blot (A) and RT‐qPCR (B, C), respectively. (D, F) Silencing of ROCK1, and the impact of IBA on the expression levels of PD‐L1 and c‐Myc in CRC cells were evaluated through western blot (D) and RT‐qPCR (E, F). (G) Schematic diagram of in vivo experiments with IBA and Y27632 treatment in mice. (H, J) Subcutaneous syngeneic mouse model of CRC, where CT26 cells were injected into BALB/c mice, including images of tumor tissue (H), tumor growth curve (I), and tumor mass (J) (*n* = 5). (K) Western blot analysis of ROCK1, c‐Myc, p‐c‐Myc, and PD‐L1 expression in mouse tumor tissues. (L) IHC analysis of ROCK1, PD‐L1, c‐Myc, and CD8 expression levels in mouse CRC tumors. Scale bar = 100 μm. (M) Positive correlation between the number of cases with high ROCK1 expression and those with high c‐Myc and PD‐L1 expression in IHC analysis of CRC patient tumor tissues. Scale bar = 50 μm. (N) Positive correlation analysis of ROCK1 expression levels with c‐Myc and PD‐L1 in IHC results of CRC patient tumor tissues. Data in (B, C, E, and F) represent the mean ± SD of three independent experiments. NS, *p* ≥ 0.05. *, *p* < 0.05.

It has been reported that ROCK1, as a threonine/serine protein kinase, can directly bind to c‐Myc, enhancing c‐Myc stability and transcriptional activity [19]. Therefore, we speculate that in CRC, ROCK1 may serve as a crucial mediator in IBA's regulation of the c‐Myc/PD‐L1 axis. In vitro assays revealed that the ROCK1 inhibitor Y27632 markedly reversed the IBA‐induced elevation of PD‐L1 protein levels, concurrently halting the IBA‐stimulated upregulation of c‐Myc and p‐c‐Myc expressions (Figure 5A). At the mRNA level, this phenomenon supported changes similar to those observed in PD‐L1 protein (Figure 5B), while alterations in c‐Myc were less apparent (Figure 5C). This aligns with our earlier conclusion that IBA predominantly regulates c‐Myc protein expression and further underscores ROCK1 as a key intermediate molecule. Utilizing lentiviral technology to silence ROCK1 in CRC cells yielded results akin to those obtained with ROCK1 inhibitors (Figure 5D–F). In vivo studies in mice further elucidated the impact of the ROCK1 inhibitor Y27632 on the downstream pathways regulated by IBA (Figure 5G), notably inhibiting the promotive effect of IBA on subcutaneous tumor growth in mice (Figure 5H–J), while also blocking the activation of downstream c‐Myc and PD‐L1 (Figure 5K,L), particularly reversing the inhibitory effect of IBA on CD8^+^ cells within the tumor microenvironment (Figure 5L). Finally, IHC analysis examined the correlation between the expression of ROCK1, c‐Myc, and PD‐L1 in CRC tissue samples. High expression of ROCK1 in CRC tissues was associated with a significant increase in c‐Myc and PD‐L1 expression (Figure 5M). Moreover, the expression levels of ROCK1 were positively correlated with those of c‐Myc and PD‐L1 (Figure 5N). In summary, IBA activates the ROCK1/c‐Myc/PD‐L1 signaling axis.

### 3.6 IBA Attenuates the Efficacy of Anti‐PD‐L1 Therapy

**FIGURE 6:**
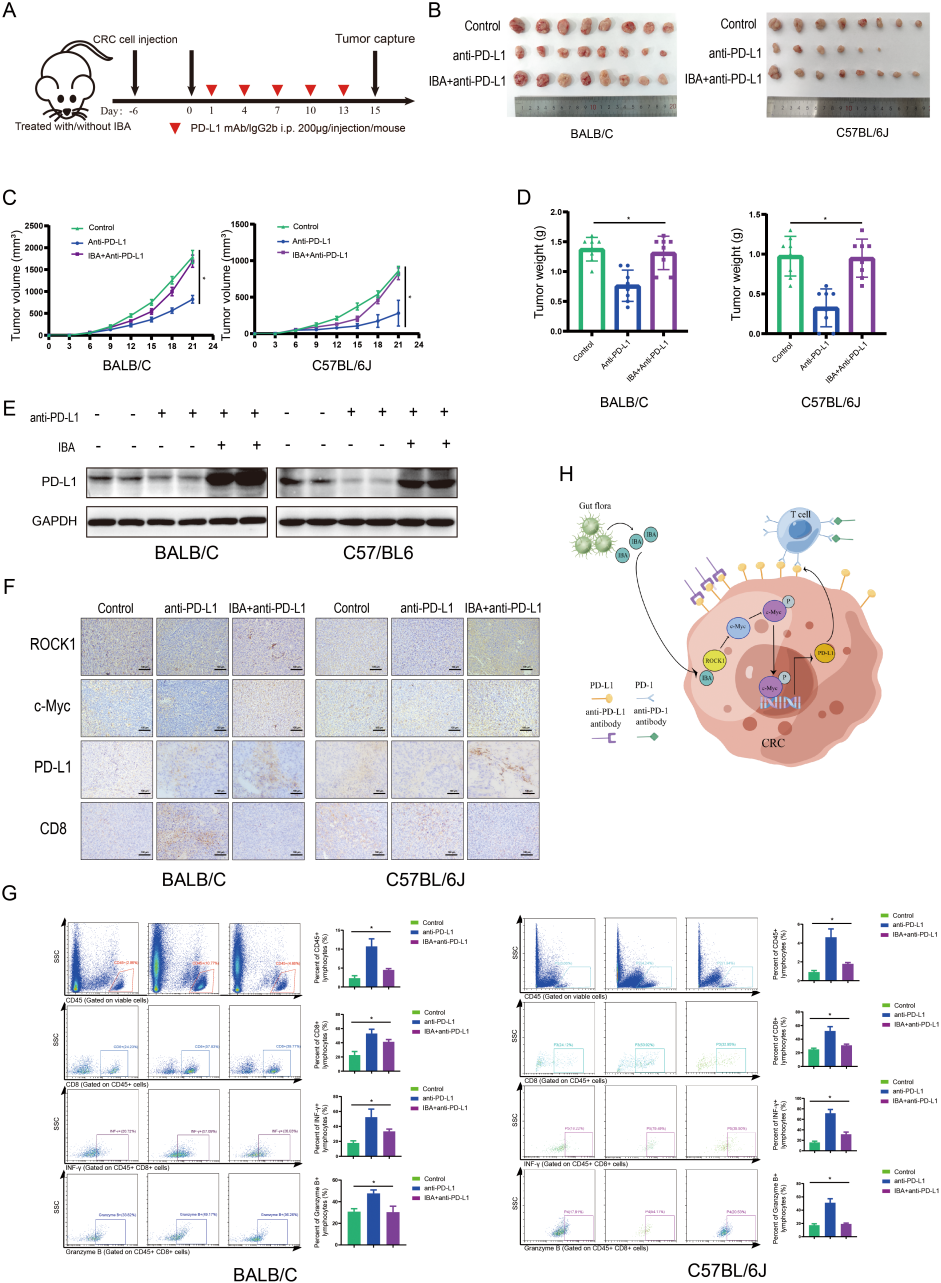
IBA attenuates the efficacy of anti‐PD‐L1 therapy. (A) Schematic diagram of the mouse model for treating CRC with IBA and anti‐PD‐L1 antibody. (B, F) Subcutaneous syngeneic mouse model of CRC, where CT26 or MC38 cells were injected into BALB/c or C57BL/6J mice, respectively, including images of tumor tissue (B), tumor growth curve (C), and tumor mass (D) (*n* = 8). Western blot analysis of PD‐L1 expression in mouse CRC tissues (E). IHC analysis of ROCK1, c‐Myc, PD‐L1, and CD8 expression in mouse CRC tissues (F). Scale bar = 100 μm. (G) Flow cytometry analysis of CD45, CD8, IFNγ, and granzyme B expression in mouse CRC tissues. (H) Schematic representation of the IBA/ROCK1/c‐Myc axis regulating PD‐L1 and its impact on the immune microenvironment in CRC. *, *p* < 0.05.

The upregulation of PD‐L1 within tumor tissues facilitates the activation of the therapeutic effects of ICIs, including anti‐PD‐1/PD‐L1 monoclonal antibodies [20, 21], while the activation of PD‐L1 may also be a factor inducing tolerance to immunotherapy [22]. Herein, we explore the impact of IBA‐induced PD‐L1 expression on the efficacy of anti‐PD‐L1 antibody therapy using a subcutaneous tumor model in mice (Figure 6A). Compared to the control group, the anti‐PD‐L1 antibody group exhibited a significant retardation in tumor growth, but this therapeutic effect was reversed by IBA (Figure 6B–D). IBA upregulated the expression levels of PD‐L1 in the anti‐PD‐L1 antibody treatment group (Figure 6E), activated the ROCK1/c‐Myc/PD‐L1 axis, and suppressed the activation of CD8^+^ cells within the tumor microenvironment (Figure 6F), reversing the improvement of TILs in mouse tumor tissues (Figure 6G). In summary, IBA diminishes the sensitivity of CRC to anti‐PD‐L1 antibody therapy.

## 4 Discussion

In this study, we have substantiated the role of IBA in promoting PD‐L1 expression in CRC, thereby reshaping the tumor immune microenvironment. The putative mechanism involves the activation of the ROCK1/c‐Myc/PD‐L1 axis, allowing tumor cells to evade immune destruction, consequently diminishing the therapeutic efficacy of anti‐PD‐L1 antibodies. Targeted disruption of IBA and its downstream signaling pathways holds promise for augmenting the immune therapeutic response in CRC (Figure 6H).

The relationship between gut microbiota metabolites and cancer has been one of the focal points of exploration in recent years. Among the tens of thousands of studies on gut microbiota, the impact of microbial metabolites on human diseases has yielded numerous significant findings [23]. Short‐chain fatty acids (SCFAs) are among the critical metabolites produced by the gut microbiota, with their highest concentrations found in the cecum and proximal colon. Appropriate levels of SCFAs can reduce intestinal inflammatory responses and provide energy for intestinal epithelial cells [24], yet aberrant expression levels of SCFAs may affect the therapeutic efficacy of CTLA‐4 antibodies in cancer patients [25]. IBA is a type of SCFA. Numerous studies have documented an aberrant increase in IBA in the feces of CRC patients [13, 26], underscoring the significance of investigating IBA's biological function in elucidating the relationship between gut microbiota metabolites and cancer. Previous research has indicated that aberrant expression of IBA promotes the metastasis of CRC. Through in vivo experiments, we discovered that IBA enhances the growth of CRC cells, increases PD‐L1 levels, and inhibits the recruitment and activation of CD8^+^ T cells. However, this phenomenon was not replicated in T‐cell‐deficient nude mouse models or in vitro cellular models, leading us to consider that the effect of IBA on tumors primarily operates through the regulation of PD‐L1 expression and immune evasion.

PD‐L1 undergoes multifaceted regulation within the organism, with current research predominantly focused on posttranslational levels. For instance, factors like SPOP, MIB2, and USP9X exert control over PD‐L1 through ubiquitination or deubiquitination [27, 28, 29], while GSK3β, AMPK, and DHHC regulate PD‐L1 via phosphorylation and palmitoylation, respectively [30, 31, 32]. In addition to posttranslational regulation, transcriptional control of PD‐L1 is equally pivotal. Research has substantiated that c‐Myc, serving as an oncogene and transcription factor, is identified as the third most amplified gene in pan‐cancer copy number analyses, playing a pivotal role in the oncogenesis of numerous human cancers and the modulation of immune responses [33]. c‐Myc is critically important for the initiation and progression of CRC, with alterations in c‐Myc commonly encountered on the pathogenesis pathway of cancer [34]. Moreover, c‐Myc can bind at the transcriptional level to the promoter region of PD‐L1, directly inducing an increase in PD‐L1 transcription [17]. Here, we report that IBA's promotion of PD‐L1 in CRC may be mediated by c‐Myc. Intriguingly, IBA does not directly bind to c‐Myc, and its effects are likely mediated indirectly.

Through the binding mass spectrometry analysis of IBA with proteins, we have identified and confirmed that IBA can bind to ROCK1. ROCK1, a member of the Rho‐associated protein kinase (ROCK) family, plays a significant role in oncogenesis [35]. Notably, ROCK1 is also a crucial binding protein of c‐Myc, engaging in direct interaction and phosphorylation of c‐Myc, which results in the stabilization and transcriptional activation of the latter [19]. Consequently, we hypothesize that ROCK1 serves as a critical molecular mediator in the upregulation of PD‐L1 by IBA, where IBA binds to and activates ROCK1, thereby triggering the downstream c‐Myc/PD‐L1 pathway and ultimately affecting the tumor immune microenvironment. Herein, we validate this mechanism and further corroborate it by analyzing the correlation among the expressions of ROCK1, c‐Myc, and PD‐L1 in CRC tissue samples.

Numerous clinical studies have corroborated that the enrichment of CD8^+^ T cells signifies a favorable prognosis for CRC patients, indicating potential benefits from ICI therapy. Conversely, a decrease in CD8^+^ T cells impedes the efficacy of ICIs [36]. At this juncture, the critical issue appears to have been identified: IBA significantly reshapes the phenotypic characteristics of the tumor immune environment, potentially rendering the response to immunotherapy uncertain. Utilizing a subcutaneous tumor model in mice, it has been confirmed that IBA reverses the significant induction of CD8^+^ T lymphocyte infiltration in CRC tissues by anti‐PD‐L1 antibody treatment and activates the suppression of IFNγ and granzyme B expression, demonstrating inhibition of CRC cell proliferation with notable therapeutic effects, consistent with previous studies [13]. When the mouse model was exposed to IBA, there was a reduction in the enhanced cytotoxic T‐cell infiltration induced by anti‐PD‐L1 antibody, a decrease in IFNγ and granzyme B expression, and significant resistance to tumor immunotherapy. Current research suggests that patients with positive PD‐L1 expression are potential beneficiaries of anti‐PD‐1 or anti‐PD‐L1 treatments. However, when PD‐L1 exceeds a certain threshold, its effect becomes contrary. For instance, overexpression of PD‐L1 induced by the aryl hydrocarbon receptor in non–small cell lung cancer reduces the efficacy of anti‐PD‐1 treatment [37]; IL‐17A increases PD‐L1 expression via the p65/NRF1/miR‐15b‐5p axis, promoting resistance of CRC to PD‐1 antibody therapy [38]. To the best of our knowledge, this is the first detailed mechanistic report on IBA's promotion of PD‐L1 expression in CRC, validated in anti‐PD‐L1 treatment.

In this study, while the IBA concentrations used were higher than those found in the peripheral blood of CRC patients [13], the stimulation of both cellular and animal models was conducted within safe limits. This design aimed to maximize the simulation of therapeutic effects. Consistent with previous studies, the use of elevated concentrations in in vitro experiments was intended to ensure a rapid and significant cellular response. Due to the absence of dynamic metabolism and bioavailability regulation in vitro, higher reagent concentrations are necessary to evaluate the full potential of IBA on PD‐L1 expression, providing a basis for dose optimization and future clinical applications [39, 40]. In animal models, achieving adequate pharmacological efficacy is similarly essential, particularly during the translation from in vitro to in vivo systems. Given the generally higher metabolic rate in mice compared to humans, elevated drug concentrations are required in murine models to mimic human pharmacodynamics [41, 42]. Moving forward, we will continue to explore the impact of varying IBA concentrations on the immunotherapy of CRC, further supporting its potential for clinical translation. Although there is much more to explore, such as investigating the impact of targeted inhibition of IBA or blocking its binding with ROCK1 on immunotherapy, or further validating the predictive value of IBA levels in clinical immune checkpoint therapy, our results demonstrate that IBA hampers the immune response to CRC immunotherapy.

In summary, our research has unveiled a novel mechanism by which IBA activates the expression of PD‐L1 in CRC. The induction of ROCK1 by IBA emerges as a pivotal factor contributing to the development of immunotherapy resistance in CRC. Upon activation by IBA, ROCK1 continually stimulates c‐Myc, thereby propelling the transcription of PD‐L1 and subsequently reshaping the immune microenvironment within tumors. Consequently, strategies aimed at inhibiting IBA or selectively blocking its interaction with ROCK1 present promising avenues for further investigation in the field of CRC immunotherapy, offering a novel therapeutic approach to overcome the challenges associated with immunotherapy resistance.

## Author Contributions

**Qiuhua Lin:** conceptualization (equal), data curation (equal), formal analysis (equal), methodology (equal), resources (equal), software (equal), validation (equal), visualization (equal), writing – original draft (equal). **Han Wang:** conceptualization (equal), data curation (equal), formal analysis (equal), methodology (equal), resources (equal), software (equal), validation (equal), visualization (equal), writing – original draft (equal). **Wenbo Chen:** conceptualization (equal), data curation (equal), formal analysis (equal), investigation (equal), resources (equal), software (equal), validation (equal), visualization (equal), writing – original draft (equal). **Xinjie Wei:** methodology (equal), visualization (equal), writing – review and editing (equal). **Jinglian Chen:** investigation (equal), methodology (equal), visualization (equal), writing – review and editing (equal). **Ying Deng:** conceptualization (equal), resources (equal), writing – review and editing (equal). **Chunyin Wei:** funding acquisition (equal), resources (equal), software (equal), writing – review and editing (equal). **Hao Lai:** data curation (equal), formal analysis (equal), visualization (equal), writing – original draft (equal). **Xianwei Mo:** project administration (equal), supervision (equal), writing – review and editing (equal). **Weizhong Tang:** project administration (equal), supervision (equal), writing – review and editing (equal). **Tao Luo:** project administration (equal), supervision (equal), writing – original draft (equal), writing – review and editing (equal).

## Ethics Statement

The study was approved by the Hospital Ethics Committee of Guangxi Medical University Cancer Hospital. All animal protocols were approved by the Animal Care and Welfare Committee of Guangxi Medical University Cancer Hospital. All patients agreed to participate in this work.

## Conflicts of Interest

The authors declare no conflicts of interest.

## Supporting information


Data S1.

## Data Availability

The article includes all data in the study, and further inquiries can be directed to the corresponding authors.
