# Supplementary material for: Isobutyric Acid Promotes Immune Evasion in Colorectal Cancer via Increased PD‐L1 Expression
Source: Cancer Med. 2024 Nov 6;13(21):e70397. doi: 10.1002/cam4.70397 (PMC11538990; doi:10.1002/cam4.70397)
Supplement: Supplementary file 1 — Data S1. [file CAM4-13-e70397-s001.docx]

**Supplemental Materials for**

**Isobutyric acid promotes immune evasion in colorectal cancer via increased PD-L1 expression**

Qiuhua Lin^1,2^**^†^**, Han Wang^1^**^†^**, Wenbo Chen^1,2^**^†^**, Xinjie Wei^1,2^, Jinglian Chen^1,2^, Ying Deng^3^, Chunyin Wei^1,2^, Hao Lai^1,2^, Xianwei Mo^1,2*^, Weizhong Tang^1,2*^, Tao Luo^1,2*^

^1^Department of Gastrointestinal Surgery, Guangxi Medical University Cancer Hospital, Guangxi Medical University, Nanning, Guangxi 530021, P. R. China.

^2^Guangxi Key Laboratory of Basic and Translational Research of Colorectal Cancer, Guangxi 530021, P. R. China.

^3^Department of Ultrasound, Guangxi Medical University Cancer Hospital, Guangxi Medical University, Nanning, Guangxi 530021, P. R. China.

^*^**Correspondence to:**

Weizhong Tang (tangweizhong@gxmu.edu.cn), Xianwei Mo (wwmmxx991@163.com), and Tao Luo (luotao@gxmu.edu.cn), Department of Gastrointestinal Surgery, Guangxi Key Laboratory of Basic and Translational Research of Colorectal Cancer, Guangxi Medical University Cancer Hospital, Guangxi Medical University, 71 Hedi Road, Nanning, Guangxi 530021, P. R. China.

**^†^** Qiuhua Lin, Han Wang, and Wenbo Chen are contributed equally to this work.

**
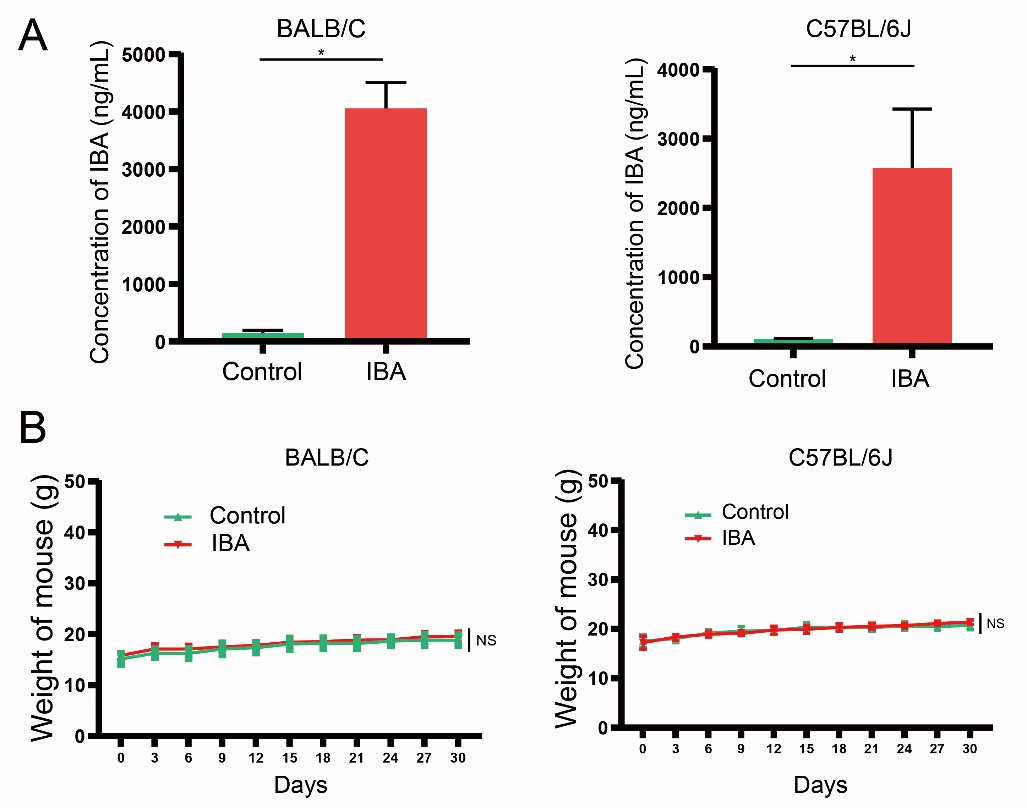
Supplementary Figures and Legends**

**Figure S1 Establishment of a high IBA mice model**

(A) LC-MS was employed to detect the concentration of IBA in serum; (B) Weight curves of BALB/c and C57BL/6J mice (n=12). NS, *p* ≥ 0.05. *, *p* < 0.05.


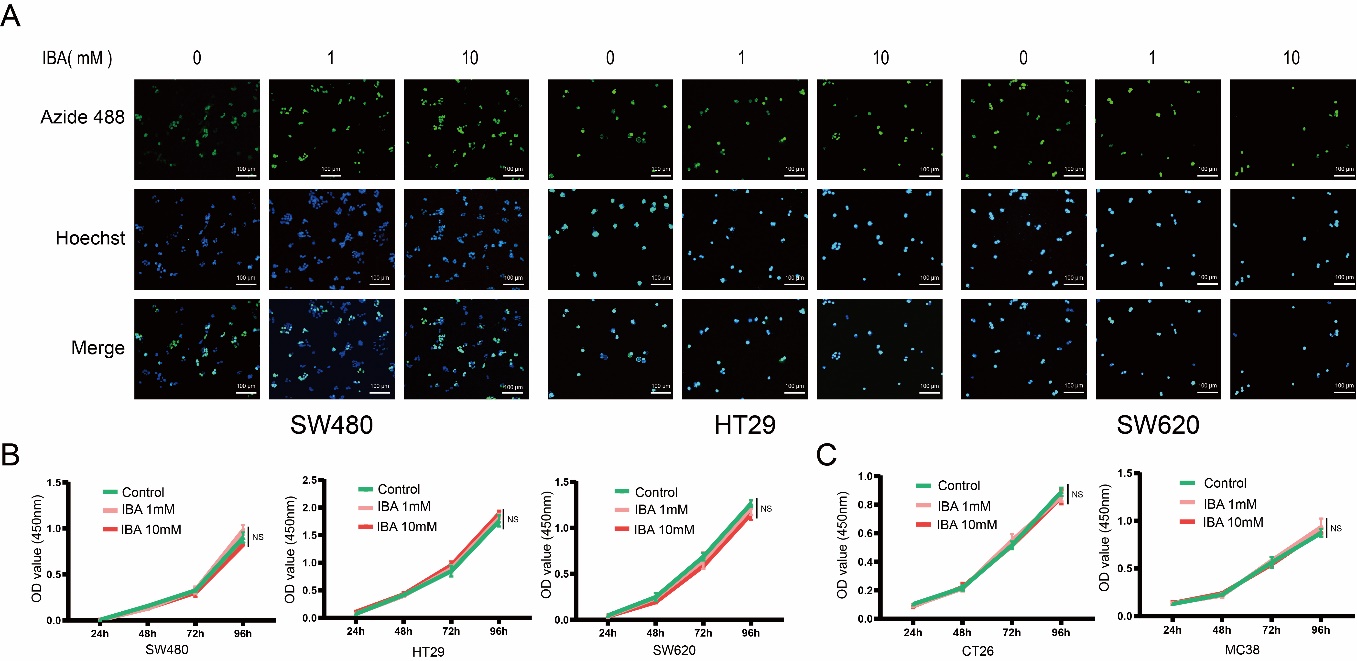


**Figure S2 The effects of IBA on the proliferation of CRC *in vitro***

(A) EdU assays were used to evaluate the effect of IBA on the proliferation of human CRC cells. Scale bar = 100 μm. (B-C) CCK-8 assays were performed to assess the effect of IBA on the proliferation of human (B) and mouse (C) CRC cells. Data in (B) and (C) represent the mean ± SD of three independent experiments. NS, *p* ≥ 0.05.


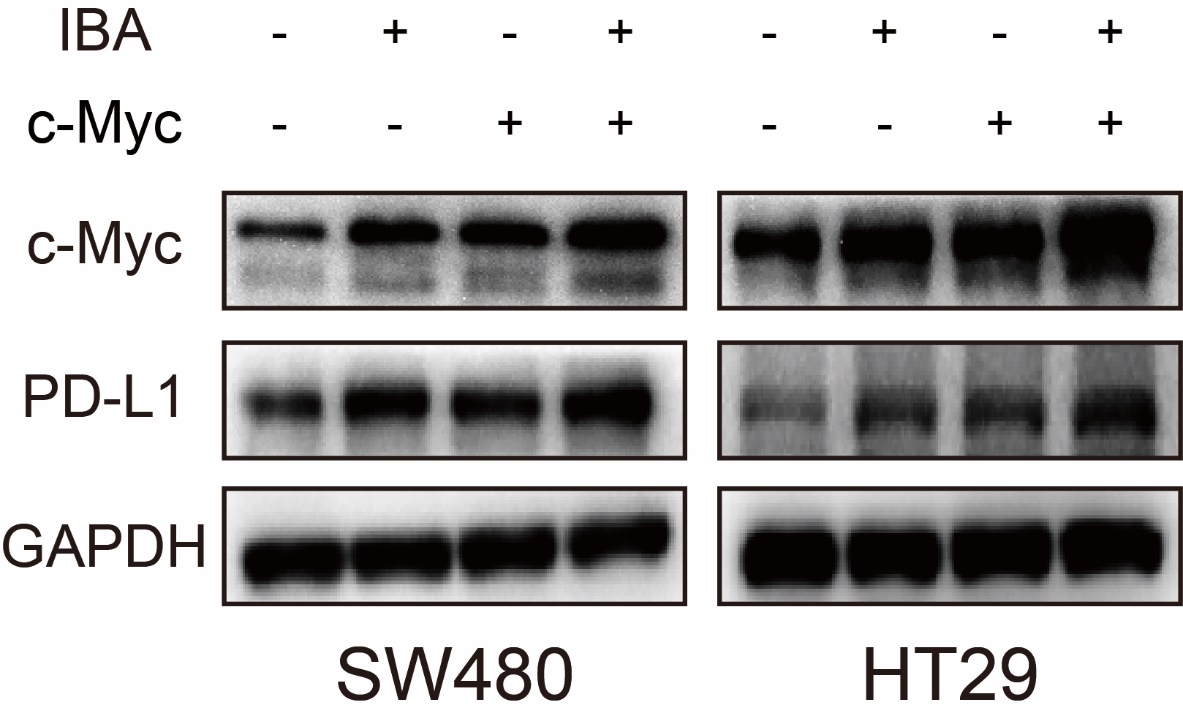


**Figure S3** CRC cells were co-incubated with or without IBA (5 mM) and c-Myc plasmid for 48 h, followed by evaluation of c-Myc and PD-L1 protein levels via Western blot analysis.

**Supplementary Table**

Table S1. Sequences of primer sets used in this study

| Genes | Primer sequences (5’ to 3’) |
| --- | --- |
| PD-L1 | F: TGGCATTTGCTGAACGCATTT |
|  | R: TGCAGCCAGGTCTAATTGTTTT |
| ROCK1 | F: AACATGCTGCTGGATAAATCTGG |
|  | R: TGTATCACATCGTACCATGCCT |
| c-Myc | F: ATGCCCCTCAACGTGAACTTC |
|  | R: GTCGCAGATGAAATAGGGCTG |
| GAPDH | F: GGAGCGAGATCCCTCCAAAAT |
|  | R: GGCTGTTGTCATACTTCTCATGG |

**Supplementary MATERIALS AND METHODS**

**Mass spectrometry analyses**

In summary, for the processes of protein elution, denaturation, reduction, and alkylation, the magnetic bead samples obtained from the in vitro pull-down assay were incubated with a reaction buffer containing SDC, TCEP, and CAA at 95°C for 10 minutes. Subsequently, the supernatant was diluted twofold with H_2_O. Trypsin (1 μg) was then added for an overnight digestion at 37°C. On the following day, peptide purification was accomplished using SDB desalting columns. The eluted peptide solution was vacuum-dried and stored at -20°C for subsequent use. For LC-MS/MS data acquisition, analysis was conducted on a Q Exactive Plus LC-MS/MS mass spectrometer equipped with a nano-electrospray source (Thermo, USA). Peptides were reconstituted in MS loading buffer (0.1% formic acid) and initially loaded onto a C18 trapping column, followed by elution onto a C18 analytical column (50 μm × 150 mm, 2 μm particle size, 100 Å pore size). A 60-minute separation gradient was employed, utilizing mobile phase A (0.1% formic acid) and mobile phase B (90% acetonitrile, 0.1% formic acid), with a constant flow rate of 300 nL/min. Data acquisition parameters included a spray voltage of 2 kV, an ion funnel RF of 40, and an ion transfer tube temperature of 320°C. For DDA mode analysis, each scan cycle comprised a full-scan mass spectrum (resolution 70 K, scan range 350 - 1800 m/z, AGC 3e6, IT 20 ms) followed by 15 MS/MS events (resolution 17.5 K, AGC 2e5, IT 50 ms). HCD collision energy was set to 28, with an isolation window of 1.6 Da, and a dynamic exclusion time of 35 s. MS raw data were analyzed using MaxQuant software (version 1.6.6), employing the Andromeda database search algorithm and MaxLFQ function. Default parameters were applied for the spectrum file search, except for using the label-free quantification mode, setting the minimum ratio count to 1, and enabling matching features across runs. Search results were filtered at a 1% FDR at both the protein and peptide levels.

**Quantification of IBA by LC-MS**

Liquid chromatography–mass spectrometry (LC-MS) was employed for the quantification of IBA. Initially, 10 mg of IBA standard substance was precisely weighed and dissolved in a 50% acetonitrile-water solution to a final volume of 1 mL, forming the standard stock solution. Subsequently, 100 μL of the individual standard solution was mixed and diluted to 1 mL, resulting in the preparation of mixed standard solution A. This solution was further diluted 50-fold to obtain mixed standard solution B. For derivatization, 40 μL of mixed standard solution B was combined with 20 μL of 200 mM 3NPH.HCL and 20 μL of 120 mM EDC.HCL (containing 6% pyridine), both dissolved in a 50% acetonitrile-water solution. The reaction mixture was incubated at 40°C for 30 minutes, followed by dilution to 200 μL with 50% acetonitrile-water and subsequent serial dilutions to various working concentrations, which were then stored in 1.5 mL EP tubes. For the analysis of mouse serum samples, 50 μL of serum was mixed with 100 μL of extraction solution (acetonitrile) and subjected to low-temperature ultrasound for 30 minutes (5°C, 40 KHz). The mixture was then centrifuged at 4°C and 13,000 rcf for 15 minutes. The supernatant was collected, to which 20 μL of 200 mM 3NPH.HCL and 20 μL of 120 mM EDC.HCL (containing 6% pyridine) were added. The reaction was carried out at 40°C for 30 minutes, followed by dilution to 750 μL with a 50% acetonitrile-water solution. The prepared samples were subsequently analyzed using an AB SCIEX QTRAP 6500+ mass spectrometer (USA). Linear regression standard curves were generated using the AB Sciex quantitative software OS, with varying concentrations of standard samples plotted on the x-axis. The peak area of the test samples was interpolated into the linear equation to calculate the concentration results.
